# Draft Genome Sequence of Vibrio vulnificus 86573B, a Bacterium Isolated from Diseased Tilapia in Taiwan

**DOI:** 10.1128/MRA.00813-18

**Published:** 2018-07-12

**Authors:** Shu-Ting Cho, Yi-Ming Tsai, Chun-Yao Chen, Chih-Horng Kuo

**Affiliations:** a Institute of Plant and Microbial Biology, Academia Sinica, Taipei, Taiwan; b Department of Life Sciences, Tzu-Chi University, Hualien, Taiwan; Loyola University Chicago

## Abstract

Vibrio vulnificus 86573B is a biotype 1 strain isolated from a moribund tilapia collected in Kaohsiung, Taiwan, during an outbreak early in 1997. Here, we report the draft genome sequence of this bacterium to facilitate the investigation of its biology and future comparative genomic analysis.

## ANNOUNCEMENT

Vibrio vulnificus is a Gram-negative bacterium found in brackish environments. This species is a known human pathogen (1) and has been associated with infections in eels (2) and tilapia (3). A Vibrio vulnificus biotype 1 strain, 86573B, was isolated from a moribund tilapia collected in Kaohsiung, Taiwan, during an outbreak early in 1997. During this outbreak, the daily mortality rate was <0.5%, which is much lower than the >1% daily mortality rates observed in the same area in the following years. To facilitate the future characterization of this strain, as well as comparative analysis with other related strains (4–6), we report here a draft genome assembly of this bacterium.

The bacterial strain was first isolated using tryptic soy agar (TSA). The purity was confirmed by multiple passages of single colonies on TSA plates. The species identification was based on 16S rRNA gene sequence typing. A sequence similarity search against the NCBI 16S rRNA database identified the type strain of Vibrio vulnificus (i.e., strain 324, NCBI reference sequence number NR_036888) as the best hit (sequence identity, 98.8%).

The procedures for whole-genome shotgun sequencing, genome assembly, and annotation were based on those described in our previous studies (6–9). Briefly, we utilized the Illumina MiSeq platform to obtain 251-bp sequencing reads from one paired-end library and one mate pair library, with approximately 321- and 215-fold coverage, respectively. The initial *de novo* assembly was performed using ALLPATHS-LG release 52188 (10). Subsequently, an iterative process was used to improve the assembly. For each iteration, all raw reads were mapped to the assembly using the Burrows-Wheeler Alignment (BWA) tool version 0.7.12 (11), programmatically checked using the MPILEUP program in SAMtools package version 1.2 (12), and visually inspected using Integrative Genomics Viewer (IGV) version 2.3.57 (13). Regions that involved possible misassembly were removed, and polymorphic sites were corrected based on the mapped reads. The annotation was performed using the Prokaryotic Genome Annotation Pipeline (PGAP) provided by the NCBI (14).

The first version of this draft genome contains 97 contigs with a combined size of 5,429,695 bp; the average G+C content is 46.4%. The annotation includes 5 rRNA genes (2 5S rRNAs, 2 16S rRNAs, and 1 23S rRNAs), 78 tRNA genes, and 4,926 protein-coding genes.

### Data availability.

This whole-genome shotgun project has been deposited at DDBJ/EMBL/GenBank under the accession number QHHR00000000. The version described in this paper is the first version, QHHR01000000.
